# Physician associate/assistant contributions to cancer diagnosis in primary care: a rapid systematic review

**DOI:** 10.1186/s12913-021-06667-y

**Published:** 2021-07-03

**Authors:** Jessica Sheringham, Angela King, Ruth Plackett, Anwar Khan, Michelle Cornes, Angelos P. Kassianos

**Affiliations:** 1 Department of Applied Health Research, UCL, 1-19 Torrington Place, 14, London, WC1E 7HB UK; 2 NIHR Cancer Awareness, Screening and Early Diagnosis Policy Research Unit, Queen Mary University of London, London, UK; 3 Waltham Forest Training Hub, London, UK; 4 NIHR Health & Social Care Workforce Research Unit, King’s College London, London, UK

**Keywords:** Physician assistants, Early Detection of Cancer, General Practice, Primary Care, primary care physicians

## Abstract

**Background:**

Symptom recognition and timely referral in primary care are crucial for the early diagnosis of cancer. Physician assistants or associates (PAs) have been introduced in 18 healthcare systems across the world, with numbers increasing in some cases to address primary care physician shortages. Little is known about their impact on suspected cancer recognition and referral.

This review sought to summarise findings from observational studies conducted in high income countries on PAs’ competence and performance on processes concerned with the quality of recognition and referral of suspected cancer in primary care.

**Method:**

A rapid systematic review of international peer-reviewed literature was performed. Searches were undertaken on OVID, EMBASE, Web of Science, and CINAHL databases (2009–2019). Studies were eligible if they reported on PA skills, processes and outcomes relevant to suspected cancer recognition and referral. Title and abstract screening was followed by full paper review and data extraction. Synthesis of qualitative and quantitative findings was undertaken on three themes: deployment, competence, and performance. Preliminary findings were discussed with an expert advisory group to inform interpretation.

**Results:**

From 883 references, 15 eligible papers were identified, of which 13 were from the USA. Seven studies reported on general clinical processes in primary care that would support cancer diagnosis, most commonly ordering of diagnostic tests (*n* = 6) and referrals to specialists (*n* = 4). Fewer papers reported on consultation processes, such as examinations or history taking (*n* = 3) Six papers considered PAs’ competence and performance on cancer screening. PAs performed similarly to primary care physicians on rates of diagnostic tests ordered, referrals and patient outcomes (satisfaction, malpractice, emergency visits). No studies reported on the timeliness of cancer diagnosis.

**Conclusion:**

This review of peer-reviewed literature combined with advisory group interpretation suggests the introduction of PAs into primary care may maintain the quality of referrals and diagnostic tests needed to support cancer diagnosis. It also highlights the lack of research on several aspects of PAs’ roles, including outcomes of the diagnostic process.

**Supplementary Information:**

The online version contains supplementary material available at 10.1186/s12913-021-06667-y.

## Introduction

In health systems such as the UK, most patients with cancer first present to primary care [1]. The role of primary care is twofold: first, to conduct investigations in primary care and second, to arrange referrals and tests conducted in secondary care [2]. Research to date has focused on primary care physicians but primary care is changing, with greater input from a range of professionals, such as physician assistants (PAs) [3–5].

PAs have recently been introduced in the UK as physician associates. Training involves an intense 2-year Masters programme, which covers similar content to medicine (e.g. anatomy, physiology, pharmacology) and provides exposure to clinical environments, including primary care. Masters graduates that pass the national Faculty of Physician Associate exams are then permitted to work as PAs throughout the UK national health service (NHS). Their role is described as complementary to doctors and involves taking medical histories, managing and diagnosing illnesses [6, 4]. PAs in the UK were not regulated as of 2021 but following a consultation in 2019 there are plans to introduce it [7]. Responsibilities should also expand when the regulatory framework in England aligns with that of other countries [7]. This will enable them to perform other tasks central to cancer recognition and referral, e.g. ordering x-rays, for which they are trained but not allowed to carry out under current regulations. Numbers of PAs in England are expected to rise significantly following recent health service reforms [8] which include funding for employment of ‘additional roles’ such as physician associates in newly established primary care networks from 2020/2021 [9].

PAs have been working in the USA (as physician assistants) for over 50 years, and 20–30% work in primary care [10]. They have been introduced in several other countries [11]. Although PAs’ roles vary both within and between health systems, may still be opportunities to learn from international experience about the impact of introducing PAs into healthcare systems.

In relation to cancer diagnosis, it is not clear whether the expansion of PA roles poses a threat or opportunity for the quality of cancer diagnostics in primary care. A systematic review of the international evidence on the role of PAs published in 2013 reported that the quality of evidence was weak with few studies comparing performance with other professionals [12]. This review, however, was undertaken over 7 years ago, and since, the volume of studies on PA performance has grown. There have been no systematic reviews examining the quality of PAs’ referral and recognition of potential cancer symptoms.

## Methods

This rapid review aimed to summarise findings from observational studies conducted in high income countries reporting on the PAs’ performance and competence on processes to support recognition and referral of suspected cancer in primary care. Specifically, it sought to answer the following research questions:

1. Which tasks relevant to cancer diagnosis in primary care conducted by PAs have been examined?
2. To what extent do these studies indicate whether PAs have the knowledge and skills to support cancer diagnosis in primary care?
3. Informed by research question 1, how does PA performance compare with performance of other clinical professionals on processes (for example investigation, history taking, referral) and outcomes of care relevant to cancer diagnosis?

Through discussion with experts in primary care, cancer diagnosis and the physician associate profession, we sought to contextualise the findings to primary care in England.

The review was registered with PROSPERO (reference: CRD42019154114) and followed PRISMA guidelines [13]. The aim was to provide timely findings in order to inform the rollout of changes in primary care in England. Therefore, streamlined methods were used which sought to maximise robustness but provide timely findings. These steps included searching only peer reviewed literature in one language; limiting double screening; focusing only on key elements of quality appraisal tools for appraisal; and integrating a knowledge translation approach into the review [14].

### Search strategy

Searches for peer-reviewed papers were undertaken on the following databases: OVID, EMBASE, Web of Science, and CINAHL. The search was conducted in November 2019 with database alerts set up to identify relevant literature published since that date. The search strategy was adapted from Skrobanski et al. to include terms related to PAs’ potential role in the diagnostic process, informed by the NHS description of typical PA tasks in England (i.e. taking medical histories from patients, performing physical examinations, diagnosing illnesses, performing diagnostic procedures, analysing test results) plus additional duties that PAs may be permitted to undertake once regulation is in place such as ordering x-rays [6, 15] (Supplementary data) Searching was limited to publications in English since 2009. While physician associates were first introduced in England before 2009, this later date was selected because access to diagnostic tools has changed considerably over the last 10 years.

Additional eligible studies were sought by hand-searching reference lists of included studies, consultation with experts and screening of citation alerts since searches were carried out.

### Study selection

#### Eligibility criteria

Inclusion criteria – piloted by two researchers on a sample of abstracts – are described in Table 1.

**Table 1:** Review eligibility criteria, highlighting changes made following piloting

**Population**	**Physician associates (PAs) or assistants working in primary care in any high-income country** [16]**.** ***CHANGE: ****The search terms initially included a range of terms for primary care settings. When articles were retrieved, several potentially relevant ones were missing. The search was amended to remove primary care terms. Instead a paper’s relevance to primary care in the UK was assessed on all retrieved records, where possible at abstract screening or otherwise at full text review.*
**Intervention/ Exposure**:	**Actions taken by PAs for patients with any symptom(s) that might be cancer** *CHANGE: Although initially planned, the search was not limited to papers with mention of possible cancer symptoms. In practice almost any symptom may be a sign of cancer so it was not feasible to turn this criterion into specific search terms. However, it was possible to exclude papers on abstract screening or full-text review where the symptom or aims of the study was clearly not relevant to cancer diagnosis,* e.g. *screening for domestic violence, care for multimorbidity.*
**Comparator:**	**Any other clinical professional** *CHANGE: Following the advice of an expert advisor we included studies where PA data were aggregated with nurse practitioners but excluded studies were PA data were aggregated with other professionals. This was in recognition of the fact that, in many settings, nurse practitioner and PA roles may overlap.*
**Outcomes**:	**Quality of symptom recognition and referral where cancer might be suspected** This comprised PA skills, confidence, performance, deployment (activities or decisions undertaken to reach a diagnosis, such as history taking, symptom recognition, referral or investigation, triage and cancer screening referral), satisfaction with care, and adherence to guidance/best practice. Excluded: Chronic disease management, non-cancer screening or primary prevention.
**Study type:**	**Peer-reviewed papers** Study design: Primary research - qualitative or quantitative Excluded: - Editorials, letters or narrative reviews - Systematic reviews though these were first searched for eligible references.

#### Screening

All identified studies underwent title/abstract and full-text screening. For title and abstract screening, a researcher independently reviewed abstracts of all studies against the inclusion criteria described above. All studies identified for inclusion underwent full-text screening. To expedite the review process, multiple reviewers split the screening between them and double screened a proportion (≥10%). Discrepancies were resolved by discussion between both reviewers and the PI.

#### Data extraction and quality appraisal

Data extraction and quality appraisal were undertaken concurrently and split across three reviewers with a proportion (10%) dual-extracted by a fourth researcher for quality assurance. A data extraction form was first piloted then used by four reviewers to extract data on during full text screening on the research question/purpose, study design, setting (clinical and geographical), sample size, sample characteristics, outcomes measured, analysis methods, results and authors’ conclusions. Appraisal focused on selected measures of methodological quality and relevance. For methodological quality, researchers considered risk of selection bias based on study descriptions of sampling strategy and response rates; and measurement bias from risks of social desirability or unvalidated measures. Studies with a high risk of bias were still described qualitatively but excluded from synthesis of quantitative results. For relevance, studies were appraised in terms of relevance to (a) UK primary care and (b) cancer diagnosis.

### Synthesis

Included studies were narratively synthesised into themes guided by the research questions:

1. PA deployment on tasks to support cancer diagnosis reported in the literature
2. PA competence and skills
3. PA performance

Quantitative and qualitative data were combined in the narrative synthesis. Quantitative findings for key outcomes were summarised from studies which compared PA with primary care physician performance and risk of bias was not high.

### Consultation and knowledge translation

An expert advisory group was convened for the project of 10 members comprising physician associates with experience of working in the UK, GPs, medical educators with PA education programmes, a patient advocate and researchers in early diagnosis of cancer. Consultation took place with the group, in the early stages of the review to identify and include any relevant literature that had not emerged from the database search. Once preliminary findings were obtained, an online meeting was held with one to one discussions for those who could not attend to discuss the interpretation and policy and research implications of the results. Themes discussed with the group focused on barriers or opportunities to maximising the contribution of PAs in England and to surface other important perceptions of PA performance and competence not identified in the literature.

## Results

### Description of included papers

**Fig. 1:**
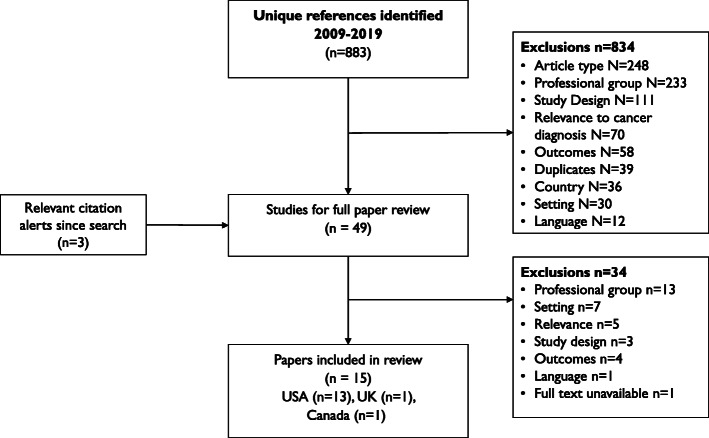
PRISMA flowchart from search to included studies

The search retrieved 883 unique records, plus three through citation alerts, of which 49 papers were included for full paper review. After excluding those not meeting inclusion criteria, 14 studies from 15 papers remained in the dataset, of which 13 were from the USA (Fig. 1). Six of the US-based studies were national. All the other studies were state-wide or regional. Ten studies pertained to primary care settings and five included both primary and secondary care (Table 2).

**Table 2:** Description of included studies

Study	Study objective	Location	Region	Setting	Sites^**a)**^	Design (data collection)	Task or outcome	Risk of bias^b)^
1. Blaes et al., 2019 [17]	Determine primary care providers’ screening practices for women at high risk of breast cancer, & examine differences in practices and knowledge of recommendations across providers	USA	Minnesota	Primary care	n/a	Cross-sectional (survey)	Screening	**High**
2. Boone et al., 2016 [18]	Understand what may limit adherence to new screening policies	USA	California	Primary care	n/a	Cross-sectional (survey)	Screening	**Medium**
3. Brock et al., 2017 [19]	Compare rates of malpractice reports and adverse actions for physicians, physician assistants (PAs), and nurse practitioners (NPs)	USA	National	Secondary & primary care	n/a	Longitudinal (claims)	Diagnosis (delay or failure to diagnose)	**Medium**
4. Burrows et al., 2020 [20]	Explore PA role integration in the Ontario healthcare system through an in-depth analysis of setting and role descriptions, described outcomes, and healthcare provider perceptions	Canada	Ontario	Secondary & primary care	19	Case study (interviews, documentary analysis)	Referrals to other physicians	**Low**
5. Drennan et al. 2015 [21]	Compare outcomes and costs of same-day requested consultations by PAs with those of GPs	England	South, East, & South West	Primary care	12	Longitudinal (medical records)	Diagnostic tests Referrals to other physicians	**Low**
6. Feeley et al., 2009 [22]	Explore how colorectal cancer screening is approached in primary care	USA	New York	Primary care	n/a	Qualitative (focus groups)	Screening	**High**
7. Hughes et al., 2015 [23]	Compare use of diagnostic tests by NPs and PAs and PCPs	USA	National	Primary care	Not stated	Longitudinal (medical records)	Diagnostic tests	**Medium**
8. Johnson et al., 2019 [24]	Determine whether Advanced Practice Providers (APPs) provided the same quality care as physicians in a variety of settings	USA	National	Secondary & primary care	4	Quasi-experimental (online vignettes)	History taking, physical examination, diagnostic tests ordered, making a diagnosis	**High**
9. Kepka D et al. 2014 [25]	Evaluate the relationship between type of medical provider seen in the past 12 months and receipt of cancer screening	USA	National	Secondary & primary care	n/a	Cross-sectional (survey)	Screening	**Medium /low**
10. Kurtzman et al., 2017 [26]	Compare the quality of care and practice patterns of NPs, PAs, and primary care physicians in health centres	USA	National	Primary care	104	Longitudinal (repeated cross- sectional surveys)	Referrals to other physicians Physical examination Reconsultation	**Low**
11. Laird et al., 2020 [27]	1) describe and compare Texas NP & PA knowledge and use of screening guidelines for colorectal cancer 2) compare recommendations for referral for genetic counselling for colorectal or endometrial cancer	USA	Texas	Primary care	n/a	Cross-sectional (survey)	Screening	**High**
12. Liu et al. 2017 [28]	What impact NPs and PAs have on utilization in a setting where physician-supervised NPs and PAs provide face-to-face primary care	USA	Georgia	Primary care	10	Quasi-experimental (medical records)	Diagnostic tests ordered Referrals to other physicians ED visits	**Medium**
13. Mafi et al., 2016 [29]	Compare APCs and physicians in providing inefficient or low-value care [radiography (for URIs and back pain), computed tomography or magnetic resonance imaging (for headache and back pain), and referrals to other physicians]	USA	National	Primary care	n/a	Longitudinal (repeated cross- sectional surveys)	Diagnostic tests ordered Referrals to other physicians	**Low**
14. Martin et al. 2020 [30]	Compare health care providers’ breast cancer screening practices for average-risk women at the ages of 40–44 and 45–49 years	USA	Minnesota	Primary care	n/a	Cross-sectional (survey)	Screening	**High**
15. Tang et al. 2016 [31]	Clinician factors are associated with prostate-specific antigen (PSA) screening in older veterans with limited life expectancy	USA	National	Secondary & primary care		Cross-sectional (medical records)	Screening	**Low/medium**

a. Sites: n/a stated where data were collected from individual providers and analysis was not available or applicable at site level

b. High, medium or low. Detailed assessment of bias and other considerations of quality and relevance are given in Supplementary data

### Study design & source of data

Thirteen studies were quantitative comprising cross-sectional (*n* = 6, surveys or medical records studies) and longitudinal designs (*n* = 7, repeated cross-sectional surveys, quasi-experimental studies and medical record cohort studies). Three studies used data from national surveys of professionals (the National Ambulatory Medical Care Survey and the National Hospital Ambulatory Medical Care Survey) [19, 21] or patients (the National Health Interview Survey with Cancer Control Supplement data on screening and HPV vaccination) [23]. Bespoke surveys were developed by adapting existing surveys, or creating new instruments, including clinical vignettes, where there was little detail about survey construction or instrument validation [24, 25].Records were obtained from national US health care records systems - Veteran Affairs [26], MediCare fee-for-service claims [28], National Practitioner Data Bank claims [29]. In two studies regional or state-wide records data were used from Kaiser Permanente’s electronic medical records in Georgia [30] and, patient records held by local, general practice systems in England [31]. There were two qualitative studies, one of which collected data via focus groups, the other used case studies and collected data through interviews and document analysis.

#### Sample

All studies included consideration of PAs’ competence and performance in relation to other primary care professionals, most commonly primary care physicians. The definition of primary care physicians varied. In the USA, primary care physicians included general practice, family practice, and internal medicine, and general practice, family medicine, internal medicine, and/or obstetrics/ gynaecology [32], whilst in England, primary care physicians were general practitioners only [21]. In five studies, PA and advanced nurse practitioner (NP) data were aggregated in the paper’s main analysis [22, 23, 25, 28, 29]. In two studies disaggregated data were available in supplementary data or secondary analyses [23, 29].

Sample sizes in surveys were between 50 [17, 17] and 4891 [20] with response rates ranging from 7.7% [21, 22] to over 80% [25]. In studies using medical records the smallest sample was 2086 [26]. In the largest study there were over 800,000 patient records but only 7% of patients received care from PAs [30]. Of the qualitative studies, Feeley et al. conducted focus groups with physicians (*n* = 56), NP/PAs (*n* = 47), and patients (*n* = 103) on colorectal screening [30]. Burrows obtained interview data from 24 PAs, and those that worked with them (17 physicians, 2 medical residents, 2 registered nurses, and 1 family health team administrator) but there was no information on the documentary sources they used [31].

#### Quality and risk of bias

Four quantitative studies had a high risk of bias due to low, unreported or biased response rates. In another quantitative study it was not possible to disaggregate NP from PA performance. These studies were excluded from the quantitative summary of study findings. Further reporting of bias and other quality/relevance concerns limiting the applicability of the study findings to this review are in the Additional file 1. We also consider the variable relevance of studies to the context of UK primary care in 2020 in Table 3 and the discussion.

**Table 3:** Applicability of review findings to current UK context. Preliminary review findings were presented remotely to the advisory group with comparisons between UK and US contexts (table) to stimulate discussion

	US	UK
History	Introduced in 1960s.	Introduced 2003. 1st UK graduates in 2007 [33].
# of PAs in primary care	Approx: 60,000 [12, 34]	In England: 359 (March 2020), up from 25 in 2015 [35].
**Regulation & powers**	· Subject to statutory regulation · Can prescribe & order ionising radiation.	· Regulation planned, not yet in place [7] · Not yet licensed to prescribe or order ionising radiation.
**Healthcare system context**	· Variable spend within & between states [5] · 76% of Americans have access · Co-payment & costs increasing [36] · Degree of gatekeeping varies between health care providers · Professionals: two models · Physicians only (family medicine, general internal medicine, general paediatrics) · Multidisciplinary team of nurses, nurse practitioners, PAs, OB/GYNs, psychiatrists, psychologists, and social workers.	· Spend determined nationally · Access almost universal · Free at point of delivery · Gatekeeping role controls access to specialist care and diagnostics · Professionals: two models · Until 2020: typically comprised general practitioners, practice nurses. · From 2020 in England: primary care networks include district nurses, PAs, physiotherapists, pharmacists, social prescribers, paramedics, podiatrists, geriatricians, social care & voluntary sector [37].
	· Acute and growing shortage of primary care physicians common to both.	
**Roles in cancer recognition & care**	· Advice on screening eligibility, organising referrals for and delivering screening tests. · Guidance varies at national, professional and state levels [30].	· Screening lies outside of primary care except for delivery of cervical screening. · National guidance for suspected cancer referrals [38].
**Training**	· Similar skills/knowledge: cancer risks, ‘red-flag’ symptoms, physical examinations [39].	
	· Similar pre-qualifying training: ~ 2 years intensive core curriculum + national exam [40].	
Themes from advisory group discussion to support interpretation of findings comprised: **Implications of ‘new’ workforce:** - PAs in the UK are a young profession. Most PAs entering primary care have little prior clinical experience so will need intensive support. However, freshly graduated, young PAs are often very ready to learn. - Many new graduates – not just PAs - feel unprepared for General Practice and initially need close clinical supervision. The ‘learning curve’ in competence may be observed for other professions too. **Regulation**: - Lack of regulation is recognised as a significant barrier to recruiting PAs into primary care. Lack of prescribing rights noted as a particular barrier. - Some PAs experienced few barriers to their own practice but noted attitudinal barriers from other staff. - Regulation may influence standing with doctors & open discussion about prescribing rights. **Other US/UK contextual differences:** - The US healthcare system is fragmented between and within states and providers. Variation in PA performance in the UK, therefore may be less variable than in the USA. - US patients have greater power to ‘shop around’ than in the UK which affects the clinician-patient power dynamic. Also, patients’ perceptions of PAs may be different to GPs which may also result in a different dynamic of PA-patient consultations. This is unexplored in the review.		

##### Findings

1. PA deployment on tasks to support cancer diagnosis

Seven studies reported on general clinical processes in primary care that would support cancer diagnosis. (Table 2) Most commonly, studies reported on ordering of diagnostic tests (*n* = 6) [21, 23, 24, 2, 17, 18]. Four studies considered referral to other physicians.^18 19 26 31^ Two studies reported on physical examinations [22, 24]. Only Johnson et al. reported on history taking [24]. Two studies explicitly linked care delivery with quality, by examining ordering of guideline-discordant tests or unnecessary referrals [24] or by scoring performance on clinical vignettes [25]. Seven studies reported on tasks related to cancer screening, specifically breast, (*n* = 3) [25, 25, 26], colorectal (*n* = 3) [26, 27, 27]) cervical (*n* = 2) [28, 29], prostate (*n* = 1) [29], and endometrial cancer (*n* = 1) [30]. (Table 2)

2. PA competence and skills

Four studies across three surveys examined practitioners’ knowledge, opinions and understanding of national guideline recommendations on cancer screening [17, 18, 30]. These studies found that, in common with nurse practitioners, PAs were more likely to recommend breast screening than physicians and both PAs and NPs had knowledge gaps on risk-stratified screening and referral for genetic counselling in adults at increased risk for colorectal cancer. All these studies, however, had a high risk of bias due to very low response rates.

In Johnson et al’s study of performance on history taking, examinations and diagnostic workup, clinicians’ responses to two clinical vignettes were scored according to their alignment with national evidence-based and system-specific recommendations. While no overall difference in PA/NP and physician performance was found, there was a lack of detail on the vignette construction and validation and it was not possible to disaggregate NP and PA performance [24].

Burrows et al’s qualitative study in Ontario compared physicians’ perceptions of PAs’ contributions in family medicine with perceptions from inpatient, outpatient and emergency settings [20]. It found experienced PAs were often valued as experts, for example: “other consulting services [such as specialist diagnostics] … started to prefer getting consults from the PA because of the PA’s understanding of the precise information that the consulting service requires” [20].

## PA performance as measured by processes and patient outcomes

Table 4 summarises quantitative findings in studies with medium or low risk of bias that compared processes and outcomes of care between PAs and primary care physicians. There were no reported differences between PAs and primary care physicians in diagnostic test ordering (3/4 analyses), referrals (3/3 analyses) or screening practices (1/1 analysis). Where differences in care processes were found (1/8 analyses), it was not possible to conclude these differences indicated better or worse quality of care. While PAs had similar rates of guideline-discordant care (PSA screening rates for older veterans with limited life expectancy) to physicians, all practitioners had higher rates than physician trainees [31].

**Table 4:** Findings from studies comparing PA performance with primary care physicians

Study: analysis	Main finding: PA performance vs primary care physicians	Patient cohort seen by PAs vs primary care physicians	Covariates included in adjusted analyses
**Diagnostic tests ordered**			
Drennan et al., 2015 [21]: Diagnostic tests (no specific ones specified)	↔ No significant difference Rate ratio 1.08 (0.89–1.30)	- Younger - From different geographical areas - Healthier/lower healthcare use	Age, acuity of presenting problem, sex, practice attendances in the previous 3 months, no. problems, chronic disease registers, socioeconomic deprivation
Hughes et al., 2015 [23]: Imaging	↑ Higher use Adjusted OR 1.34 (1.27–1.42) ^a)^	- Younger - higher % female - higher % of white ethnicity - Healthier - From different geographical areas	Patient age group, sex, race, state, urban, comorbidity.
Kurtzman et al., 2017 [26]: Imaging	↔ No significant difference Adjusted OR 1.14 (0.84–1.54)	Similar (age, gender, ethnicity, payer source) to PCPs.	Age, sex, race, ethnicity, payer, metro status, region, reason for visit, health centre type, education, year.
Mafi et al., 2016 [29] - Radiography (in ‘low value’ cases) ^b)^	↔ No significant difference 10.2 in PCP vs 11.4 in PAs (alone), *p* = 0.71 and 9.5% in PAs (shared) *p* = 0.75	- Younger - From different geographical areas	Patient age, sex, race or ethnicity, comorbidity, symptom acuity, insurance status, urban location, geographic region, year
- CT or MRI (in ‘low value’ cases)	↔ No significant difference 6.0 in PCP vs 9.9 in PAs (alone), *p* = 0.3 and 6.8% in PAs (shared) *p* = 0.69		
**Referrals to other physicians**			
Drennan et al., 2015 [21]	↔ No significant difference Rate ratio 0.95 9 (0.63–1.43) *p* = 0.80	- Younger - From different geographical areas - Healthier/lower healthcare use	Age, acuity of presenting problem, sex, # practice attendances in the previous 3 months, # problems, # chronic disease registers, socioeconomic deprivation
Kurtzman et al., 2017 [26]	↔ No significant difference Adjusted OR 1.17 (0.87–1.56)	PAs saw similar patient profile (age, gender, ethnicity, payer source) to PCPs.	Age, sex, race, ethnicity, payer, metro status, region, reason for visit, health centre type, education, year.
Mafi et al., 2016 [29]: Situations in which referral considered to be low value	↔ No significant difference 8.2 in PCP vs 5.9 in PAs (alone), *p* = 0.52 and 8.6% in PAs (shared) *p* = 0.86	- Younger - From different geographical areas	Patient age, sex, race or ethnicity, comorbidity, symptom acuity, insurance status, urban location, geographic region, and year
**Screening**			
Tang et al., 2016 [31]: PSA screening rates for patients with limited life expectancy	↔ No significant difference c) Screening offered in 41.3% of cases by PAs vs 41.5% by PCPs	Not reported	Patient age, race, marital status, income, education, clinician clustering
**Outcomes**			
Brock et al., 2017 [19]: Malpractice reports per 1000 clinicians ^d):^ diagnosis related claims comprise diagnosis failure or delay in diagnosis	↓ Lower payments Physician median payments ranged from 1.3 to 2.3 times higher than PAs or NPs	No data but differences in breadth of patient acuity proposed as possible explanation for findings.	n/a
Drennan et al., 2015 [21] - Re-consultation within 14 days for the same or a linked problem - Patient Satisfaction ^e)^	↔ No significant difference Adjusted rate ratio 1.24 (0.86–1.79), *p* = 0.25	- Younger - From different geographical areas - Healthier/lower healthcare use	Age, acuity of presenting problem, sex, # practice attendances in the previous 3 months, # problems, # chronic disease registers, socioeconomic deprivation
	↔ No significant difference Adjusted rate ratio 1.00 (0.42–2.36), *p* = 0.99		
Kurtzman et al., 2017 [26]: Re-consultation	↔ No significant difference Adjusted odds ratio 0.77(.52–1.13)	PAs saw similar patient profile (age, gender, ethnicity, payer source) to PCPs.	Age, sex, race, ethnicity, payer, metro status, region, reason for visit, health center type, education, year.

a. Hughes:. In the main analysis, nurse practitioner and PA data were aggregated as APC. In sensitivity analyses: NPs ordered less imaging than PAs (OR, 0.59 [0.53–0.66]); APCs ordered less imaging than PCPs for acute respiratory tract infection (OR, 0.68 [0.51–0.90]); Differences were greater for radiography than non-radiography imaging

b. Mafi: Findings were presented for both hospital and office based primary care settings. 89.9% of the data reflected visits to clinicians in office-based physician practices (data from the NAMC), so these figures are presented. Disaggregated data from supplementary data are presented here. Alone reflects visits to PAs where they saw the patient without a physician; shared reflects consultations where a physician was alongside

c. Tang: Men whose clinician was a physician trainee had substantially lower PSA screening rates than those with an attending physician, nurse practitioner, or physician assistant

d. Brock: Diagnosis malpractice claims, while higher for physicians, comprise a greater proportion of PA than physician claims (53% vs 32%). This result may be partially explained by the presence of surgeons and anaesthesiologists in the physician group, or it may signal where PAs and NPs might be most at risk for error

e. Drennan: other findings comprised: consultation duration was longer for PAs than GPs but costs per consultation were lower

Three studies reported patient outcomes. These comprised re-consultation rates in primary care [21, 26], satisfaction and malpractice claims [19], with a brief breakdown of claims due to diagnostic failure or delays in diagnosis [19], but no studies reported on the timeliness of cancer diagnosis (e.g. stage, survival). There were no reported differences in general patient outcomes (satisfaction, re-consultation rates). While PAs had fewer malpractice payments than physicians, a greater proportion were related to diagnosis. As noted by the authors, it may signal that PAs might be at greater risk of diagnostic error but could also be explained by the presence in the physician group of surgeons and anaesthesiologists -who had malpractice claims related to surgical outcomes [19].

Where it was reported, the profile of patients seen by PAs differed from that seen by primary care physicians in all but one study.

## Discussion

### Summary

This review of peer-reviewed literature combined with advisory group interpretation suggests the introduction of PAs into primary care may maintain the quality of referrals and diagnostic tests needed to support cancer diagnoses. It also highlights the lack of research on several aspects, particularly across the range of countries where PAs are deployed and on the outcomes of the diagnostic process.

### Strengths and limitations

This review, the first of its kind to focus on cancer care, provides some timely insights into the contribution of PAs in an important sphere of activity that may inform the expansion of the physician associate profession in England. It also addresses some of the limitations of the last major systematic review in 2013 examining the contribution of PAs to primary care, which reported the quality of evidence was weak with few studies comparing performance with other professionals [12]. There are important limitations, however.

Most (13/15) studies came from USA, which limits the transferability of findings to other healthcare systems. In particular, in the US the role of primary care professionals in cancer diagnosis may be different; they are not always required for referral to specialists but they are often central in organising cancer screening (a task led by cancer screening hubs in England). Studies undertaken in other countries (Netherlands, Israel, Germany) were identified but excluded because in these studies PAs were not deployed in primary care settings. However, eligible studies from the UK and Canada – where access to specialist care is normally via a family physician [41] - provided corroborative and complementary insights to those from USA. Moreover, US-based studies have relevance internationally for two other key reasons. Firstly, the drivers for the introduction of PAs have been experienced globally, i.e. shortages in primary care providers amid increasing patient demand, and shifts to multidisciplinary models of primary care teams to provide care [3]. Secondly, they give some indications of how PAs that are regulated and integrated into the healthcare system might perform on processes such as ordering of rx-rays that are not currently permitted in the UK.

None of the studies sought specifically to investigate the effect of PAs on cancer diagnosis. Some excluded cases with ‘red flag’ symptoms which might exclude cases where cancer was suspected. However, red-flag symptoms are present in only a minority of cancer diagnoses, and UK guidance specifically recommends investigation of a wide range of symptoms [38]. Five studies presented only aggregated data for NPs and PAs. Numbers of PAs may be smaller than NPs, so there is a risk that findings are driven by NPs rather than PAs. This aggregation, therefore, may miss important differences in care. Where sub-analyses had disaggregated data, PAs data was often more similar to primary care physicians than NPs. To inform workforce decisions in future studies, PA and NP performance need to be reported separately.

Most studies considered PAs’ performance from the perspective of other clinical professionals only; views of patients and non-clinical practice staff were absent from 12/15 studies. As others have reported, patients are open to seeing PAs and experience with them is largely positive when the role is explained [42, 43]. Studies so far have focused on preferences and degree of satisfaction with PAs. As Table 3 comments indicate, given the potential difference in status and duration of training, patients may develop a different relationship, and communicate in different ways with PAs than with primary care physicians.

The streamlining of review methods did result in findings within a relatively short period of time that were shared to influence practice. Streamlining review methods may have resulted in missing relevant papers, particularly due to narrowness of the search (restricted to English paper and since 2009). However, a systematic review conducted in 2013 identified major gaps in the literature at this point so extending the search to find papers published earlier than 2009 would be unlikely to yield further insights.

#### Comparison with existing literature

Our principal finding – that in most studies PAs performed similarly to physicians – is largely in line with findings from other studies [12]. In the UK a suite of studies examining the impact of PAs in primary care at micro, meso and macro levels in 2014 reported PAs were acceptable, effective and efficient in complementing the work of GPs [21, 35, 44]. At this time, however, there were just 25 PAs working in primary care, with around half trained outside of the UK, which may limit the transferability of this study to a context where most PAs have been trained in the UK and their presence is the norm, not the exception. As others have noted, this finding does not mean that PAs and physicians deliver equivalent care in general. Indeed, in common with other studies, the profile of patients seen by PAs often differed from those of primary care physicians, and generally seemed to be healthier [44]. The findings may indicate, however, that there are circumstances in which the additional clinical acumen amongst primary care physicians gained by more training and experience may not be required [45].

In common with the wider literature, this review also highlighted that PAs’ deployment varied between (and within) settings [20, 35, 46]. Lack of regulation and prescribing rights, is understood as a significant barrier to expanding their role in the UK [46]. However, aside from regulation, there are other barriers to delegation. In particular, there is evidence of some resistance and hostility from other health care professionals where there is perceived role overlap or competition for training opportunities [47]. This resistance appears to lessen when there is greater understanding of the role [47]. For PA skills to be utilised appropriately, the whole primary care team need to be clear about and accept the role of PAs in their setting. This role clarity is also required by non-clinicians also to ensure that patients are triaged to the most appropriate clinician [48, 49].

## Conclusions and implications for research, policy and practice

This review suggests that the expansion of PAs working in primary care may maintain the quality of care needed to support cancer diagnosis in high income settings. This is important, given concerns that PAs might provide poorer quality of care [20, 24, 28, 29]. It is also important to guide deployment of PAs in contexts like the UK, where their roles could be expanded to cover tasks like ordering of X-rays following regulation. The review also highlights important gaps in the evidence base, particularly the lack of research from settings outside of USA and how primary care workforce changes may affect the timeliness of cancer diagnosis. For research to explore the impact of new professions on the timeliness of diagnosis, amendments  to research and monitoring are needed to collect data on consultations with a range of professionals other than physicians.

Although we discovered no adverse outcomes from the introduction of PAs, it is clear that PAs need to be actively integrated into their working environments. Integration of PAs may require strategies for the whole practice. For example, support for clinical supervisors could enable them to maximise safe delegation to PAs. Support to primary care leaders could promote PAs’ integration into wider team, through clarifying respective clinical roles.

The context of primary care has altered significantly since the studies in this review were conducted. International guidance on the role of primary care in cancer acknowledges the planned structural shift away from a model of the lone practitioner, but provides no insight into the potential role of PAs [3]. Further studies should examine the impact of emerging professions such as PAs on timely cancer diagnosis in this new context of primary care.

## Supplementary Information

**Additional file 1.**

## Data Availability

All data are to be found in the publications references in the article.
