# Supplementary material for: Physician associate/assistant contributions to cancer diagnosis in primary care: a rapid systematic review
Source: BMC Health Serv Res. 2021 Jul 3;21:644. doi: 10.1186/s12913-021-06667-y (PMC8254243; doi:10.1186/s12913-021-06667-y)
Supplement: Supplementary file 1 — Additional file 1. [file 12913_2021_6667_MOESM1_ESM.docx]

**Physician associate/assistant impacts on cancer diagnosis in primary care: A systematic review**

**AUTHORS**

Jessica Sheringham, Department of Applied Health Research, UCL, 1-19 Torrington Place, London, WC1E 7HB, UK

Angela King, NIHR Cancer Awareness, Screening and Early Diagnosis Policy Research Unit, Queen Mary University of London

Ruth Plackett, Department of Applied Health Research, UCL

Anwar Khan, Director of Research & Education, Waltham Forest CCG

Michelle Cornes, NIHR Health & Social Care Workforce Research Unit, King’s College London

Angelos P Kassianos, Department of Applied Health Research, UCL

**Corresponding author:** Jessica Sheringham

Department of Applied Health Research, UCL, 1-19 Torrington Place,

14 London WC1E 7HB. Phone: +44 7919444064

Email: j.sheringham@ucl.ac.uk

**MeSH terms:** Physician assistants; Early Detection of Cancer; General Practice; Primary Care; primary care physician**s**

## **Supplementary data**

### Search strategy

V 1.6

Run on OVID, 11 November 2019, repeated on EMBASE, WoS, and CINAHL.

Database: Ovid MEDLINE(R) and Epub Ahead of Print, In-Process & Other Non-Indexed Citations and Daily <1946 to September 30, 2019>

--------------------------------------------------------------------------------

1 diagnosis/ or clinical decision-making/ or delayed diagnosis/ or diagnostic errors/ or diagnostic imaging/ or diagnostic techniques, cardiovascular/ or diagnostic techniques, digestive system/ or diagnostic techniques, respiratory system/ or diagnostic tests, routine/ or mass screening/ or medical history taking/ or physical examination/ or symptom assessment/ or early diagnosis/ or incidental findings/ (299333)

2 Mammography/ (28995)

3 (digital rectal examination or prostate specific antigen test or PSA test).tw. (4747)

4 (rectal palpations or rectal palpation or palpation rectal examinations or digital rectal examination or digital rectal or digital rectal examinations or dre).mp. [mp=title, abstract, original title, name of substance word, subject heading word, floating sub-heading word, keyword heading word, organism supplementary concept word, protocol supplementary concept word, rare disease supplementary concept word, unique identifier, synonyms] (6665)

5 (((fecal or faecal) and (immunochemical or occult)) or FOBT).tw. (4958)

6 immunoassay/ or enzyme-linked immunosorbent assay/ or enzyme multiplied immunoassay technique/ (173008)

7 (fecal or faecal).tw. (78349)

8 6 and 7 (2016)

9 5 or 8 (6904)

10 (screening* or screen* or diagnos* or detect* or work up or assessment or evaluation or workup or recognise or recogniz*).tw. (6250063)

11 "Referral and Consultation"/ (64012)

12 (consultation* or clinical decision making or clinical reasoning or clinical judgement or clinical consulting).tw. (91126)

13 (endoscop* or colonoscop* or sigmoidoscop* or mammogra*).tw. (247298)

14 (xray or x ray or x-ray).tw. (311894)

15 (history taking or referral* or physical examination).tw. (161318)

16 (computed tomograph* or CT scan or ct scan or computerized tomography).tw. (305186)

17 early diagnosis/ or "early detection of cancer"/ (47177)

18 ((detect* or diagnosis) and earl*).tw. (431217)

19 (endoscopy or sigmoidoscopy or colonoscopy or mammography or x ray).sh. (107613)

20 1 or 2 or 3 or 4 or 5 or 6 or 7 or 8 or 9 or 10 or 11 or 12 or 13 or 14 or 15 or 16 or 17 or 18 or 19 (7073977)

21 physician assistant {Including Related Terms} (5653)

22 Physician Assistants/ (5437)

23 ((assistant or associate) and physician).tw. (2282)

24 ((medical or doctor*) and assistant).tw. (3013)

25 (feldsher or physician extender or physician's extender).tw. (434)

26 21 or 22 or 23 or 24 or 25 (9138)

27 20 and 26 (419)

28 (sign or symptom or red flag).tw. (274091)

29 (cancer or malignan*).tw. (1958447)

30 33 or 34 (2205746)

1. 27 and 30 (54)

## Risk of bias and other concerns that limit studies’ applicability to this review

During critical appraisal, limitations in study design and conduct were recorded. Studies deemed to have a high risk of bias were excluded from a quantitative summary of study findings.

| **Auth, date** | **Study objective** | **Location** | **Risk of bias (High, Medium/low):** notes | **Other significant quality/ relevance limitations** |
| --- | --- | --- | --- | --- |
| 1. Blaes, et al, 2019 | Determine primary care providers’ breast cancer screening practices for women at high risk of breast cancer, & examine differences in practices and knowledge of recommendations across provider characteristics | USA | **High**: very low (7.7%) response rate from PAs | **Relevance**: screening responsibilities in primary care in UK and USA differ |
| 1. Boone, et al, 2016 | Understand what may limit the adherence to new screening policies | USA | **Medium**: low (28%) response rate from PAs | **Relevance**: screening responsibilities in primary care in UK and USA differ |
| 1. Brock, et al, 2017 | Compare rates of malpractice reports and adverse actions for physicians, physician assistants (PAs), and nurse practitioners (NPs). | USA | **Medium**: Unknown whether errors reflect actions of an individual or team |  |
| 1. Burrows et al, 2020 | Explore PA role integration in the Ontario healthcare system through an in-depth analysis of setting and role descriptions, described outcomes, and healthcare provider perceptions | Canada | **Low:** clear description & justification of study sample limited non-physician and no patient input | **Relevance**: limited to services that employed PAs, so uninformative about sites that decided not to employ them |
| 1. Drennan, et al 2015 | Compare outcomes and costs of same-day requested consultations by PAs with those of GPs | England | **Low:** clear inclusion criteria to justified sample size |  |
| 1. Feeley, et al, 2009 | Explore how colorectal cancer (CRC) screening is approached in primary care. | USA | **High**: 9% response rate from participants to study invitation | **Relevance**: screening responsibilities in primary care in UK and USA differ |
| 1. Hughes, et al, 2015 | Examine the use of diagnostic tests— specifically medical imaging—by APCs as a directly observable and quantifiable measure for comparing the care they provide with that of PCPs | USA | **Medium** (classification): under some circumstances work performed by APCs is coded by their supervising physician, which may underestimate differences between APCs and physicians |  |
| 1. Johnson et al, 2019 | Determine whether Advanced Practice Providers (APPs) provided the same quality care as physicians in a variety of settings | USA | **High**: small and uncertain number of PAs in the study and response rates not reported |  |
| 1. Kepka D et al 2014 | Evaluation of the relationship between type of medical provider seen in the past 12 months and receipt of cancer screening | USA | Med/Low: reliant on patient self-report but high response rate | **Relevance:** findings reported aggregating PA/PCP – not possible to compare services provided by APRNs OR PAs with those provided by PCPs |
| 1. Kurtzman, et al, 2017 | Compare the quality of care and practice patterns of NPs, PAs, and primary care physicians in health centres. | USA | **Low**: reliant on practitioner self-report but high response rates and results largely unchanged in sensitivity analyses |  |
| 1. Laird et al, 2020 | 1) To describe and compare Texas Nurse Practitioners’ and Physician Assistants’ knowledge and use of current screening guidelines for individuals at varying risk for colorectal cancer (2) To compare their recommendations for referral for genetic counselling for persons at increased risk for colorectal or endometrial cancer | USA | **High**: 7.4% response rate, unknown whether respondents were representative of all PAs/NPs | **Relevance:** screening responsibilities in primary care in UK and USA differ |
| 1. Liu, et al 2017 | What impact NPs and PAs have on utilization in a setting where physician-supervised NPs and PAs provide face-to-face primary care | USA | **Medium**: ecological analysis – unclear how PAs/NPs worked with PCPs in different models | **Relevance:** Not possible to disaggregate NP and PA data |
| 1. Mafi, et al, 2016 | Compare APCs and physicians in providing inefficient or low-value care [radiography (for URIs and back pain), computed tomography or magnetic resonance imaging (for headache and back pain), and referrals to other physicians] | USA | **Low**: reliant on practitioner self-report but high response rates |  |
| 1. Martin, et al 2019 | Compare health care providers’ breast cancer screening practices for average-risk women at the ages of 40–44 and 45–49 years and | USA | **High**: very low (7.7%) response rate from PAs | **Relevance:** screening responsibilities in primary care in UK and USA differ |
| 1. Tang, et al 2016 | Clinician factors are associated with prostate-specific antigen (PSA) screening in older veterans with limited life expectancy | USA | **Low/medium**: records based study so unclear what % of data were excluded that did not meet inclusion criteria and it is possible some PSA tests included in the study were conducted for non-screening purposes | **Relevance**: screening policies & responsibilities in primary care in UK and USA differ. |
